# A mechanical and simplified model for RC elements subjected to combined shear and axial tension

**DOI:** 10.1038/s41598-022-11577-y

**Published:** 2022-05-12

**Authors:** A. Deifalla, F. M. Mukhtar

**Affiliations:** 1 Department of Structural Engineering and Construction Management, Future University in Egypt, New Cairo City, 11835 Egypt; 2 Department of Civil and Environmental Engineering, King Fahd University of Petroleum & Minerals, Dhahran, 31261 Saudi Arabia; 3 Interdisciplinary Research Center for Construction and Building Materials, King Fahd University of Petroleum & Minerals, Dhahran, Saudi Arabia

**Keywords:** Engineering, Civil engineering

## Abstract

Very little is known about the shear behavior of elements, in particular those subjected to axial tension. The shear accompanied by tensile forces could cause premature failure of reinforced concrete, which is sudden with minimal warning. Therefore, understanding the shear behavior of reinforced concrete (RC) elements, including those subjected to axial tension, is an ultimate goal of the worldwide research community. In the current study, a new shear mechanical model for RC elements subjected to axial tension is developed, which makes physical sense and explains the behavior. The model is strain-based, inspired by the critical crack theory model (CSCT). In addition, the proposed model extended CSCT (ECSCT) quantifies the effect of axial tension forces on the shear strength in terms of reduction in the compression zone depth and increase in the longitudinal strain. Moreover, the nonlinear trend observed in the literature was implemented using nonlinear multi-variable regression. The ECSCT is validated and compared with available design methods with respect to an extensive database, including 180 elements tested under shear and tension from 18 different research investigations. The ECSCT provided an accurate and physically sound model yet safe to an acceptable extent. Last but not least, a simplified model for the purpose of design is proposed. The simplified model was chosen based on the mechanical model and calibrated using the extensive experimental database. The simplified model provided an accurate and simple model, yet safe to an acceptable extent.

## Introduction

**Figure 1:**
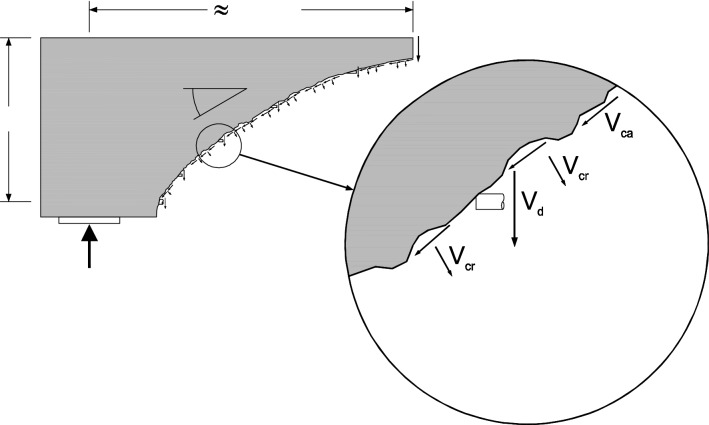
Shear failure mechanisms^27^.

Shear failure of reinforced concrete (RC) elements is sudden and should be carefully considered, in particular, those without stirrups or with stirrups^1–21^. In addition, RC elements subjected to shear combined with axial tension are still a dilemma^22–26^, which occurs in many situations. Many studies have been conducted to understand the effect of axial tension on the shear strength of RC. However, the physical significance of the tensile force on the shear design is not well defined yet. Shear behavior of RC elements is a complex problem that involves many mechanisms, as shown in Fig. 1^27^. Those mechanisms include but are not limited to: (1) direct shear through the compression zone, (2) friction along the sides of the diagonal shear cracks, (3) dowel action through longitudinal reinforcements crossing the diagonal cracks, (4) residual tensile stresses transferred across the diagonal, (5) the aggregate interlock across the diagonal crack. Suppose the beam is subjected to tensile axial forces, the crack width and the longitudinal strain increase. On the other hand, the compression zone depth, the residual tensile strength, and the aggregate interlock decrease. Recently, Deifalla^26^ recommended using the observed behavior to improve and simplify the current physically sound-based models. The current study aims to develop and propose a mechanical model for RC elements under combined shear and tension. Inspired by the critical shear crack theory^28^, a mechanical model named extended critical shear crack theory (ECSCT) was developed to include the effect of tensile forces. The strength of the experimental database was calculated using the proposed model and compared with existing design codes. In addition, a simplified model was proposed, which was found to be better for design purposes.

## Recent findings

For the last seven decades, several pioneering studies^26,29–33^ have been conducted to investigate the shear strength of elements under axial tensile forces; a brief recount of the most recent findings is as follows.

**Figure 2:**
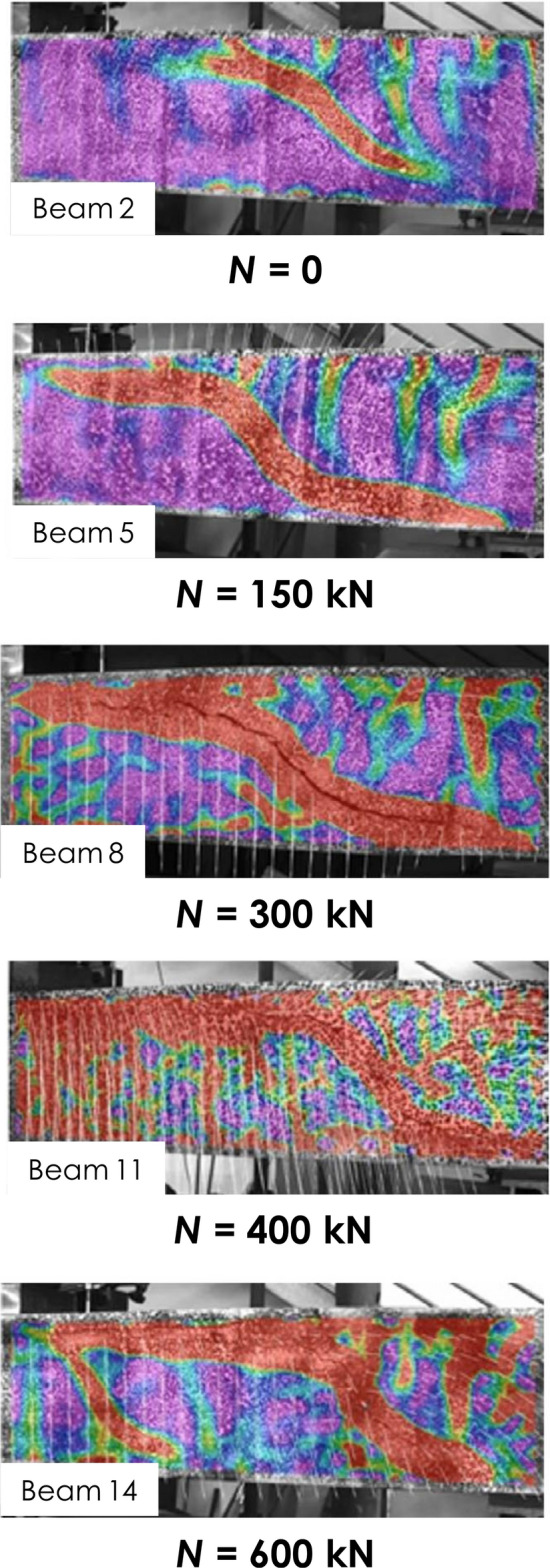
Experimentally observed behavior of RC beams under combined shear and tension^31^.

**Figure 3:**
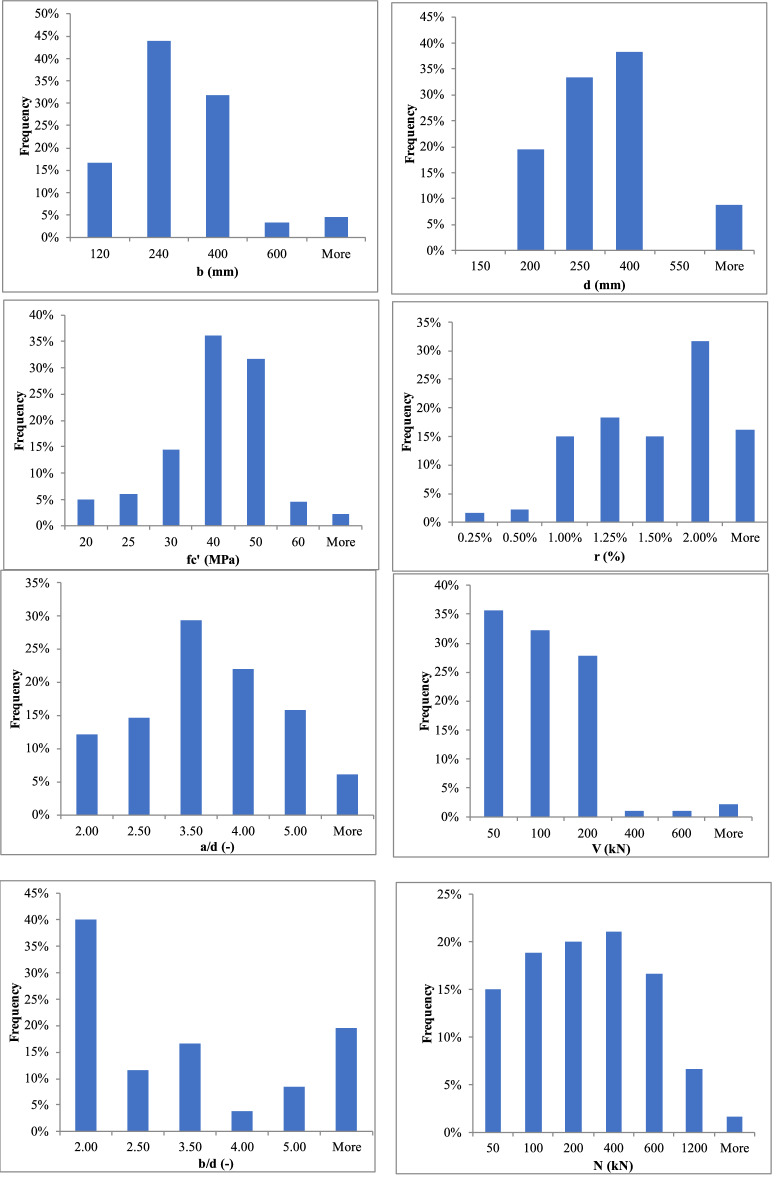
The profile of data base.

- The significant variables that affect the shear strength are element dimensions, reinforcement configuration, loading configuration, and boundary conditions; thus, the conclusions of different studies were inconsistent with each other^31^. For example, the device used to apply the axial tension could cause accidental restraint at the ends of the tested element^33^.
- The angle of inclination of shear cracking is significantly affected by the axial tension, which makes it steeper^33^. On the other hand, the shear strength of beams with well-detailed longitudinal reinforcements is not affected by axial tension. This is due to the aggregate interlock mechanism. In addition, the compression longitudinal steel reinforcements decrease the effect of axial tension forces on shear, if any^29^.
- A nonlinear relationship between the axial everyday tensile stresses and the shear strength was found by several researchers^32^. This contradicts the long-standing linear relation implemented by both the ACI^34^ and the EC2^35^.
- Pham^31^ observed a decrease in the compression zone depth with the increase in the axial tensile forces, as shown in Fig. 2.
- In general, design codes, including but not limited to the ACI and the EC2, are overly conservative, especially for cases of high tensile forces^26^.
- An extensive experimental database of elements tested under shear and tension was gathered, combining the database complied by Deifalla^26^ and Ehmann^30^. A total of 180 elements from 17 different research investigations. The data covered a wide range of all influential variables, as shown in Table 1 and Fig. 3^30–33,36–49^. The effective parameters included the axial tension, the size, the shear-span to depth ratio, the concrete strength, flexure reinforcement ratio, and the width to depth ratio were gathered.

**Table 1:** Experimental database.

References	Label	d (mm)	b (mm)	fc′ (MPa)	$\rho$ (–)	fy (MPa)	a/d (–)	N (kN)	V (kN)
^36^	9	284	175	23	0.41%	343.4	3.69	86	19.68
	10	284	175	23	0.41%	343.4	3.69	68	24.13
^37^	4	254	152	46	1.03%	399.9	3.00	29	44.48
	5	254	152	16	2.07%	399.9	3.00	29	33.36
	11	254	152	15	3.10%	399.9	3.00	61	42.26
	16	254	152	30	1.03%	399.9	5.40	48	28.02
	19	254	152	19	2.07%	399.9	5.40	29	40.03
	20	254	152	48	2.07%	399.9	5.40	29	57.83
	21	254	152	51	2.07%	399.9	5.40	61	56.93
	23	254	152	19	3.10%	399.9	5.40	29	42.26
	25	254	152	28	3.10%	399.9	5.40	48	51.15
	26	254	152	29	1.00%	399.9	5.40	80	42.26
	29	254	152	53	3.10%	399.9	5.40	29	66.72
^38^	A1T	381	178	28	3.78%	517.3	2.50	144	122.55
	C1T	381	178	29	3.78%	517.3	3.38	144	120.21
	J1T	381	178	29	3.78%	517.3	2.50	144	87.00
^39^	N3	272	152	33	1.46%	427	2.80	120	42.00
	N4	272	152	34	1.46%	427	2.80	90	42.00
	N5	272	152	32	1.46%	427	2.80	60	48.00
	N6	272	152	32	1.46%	427	2.80	70	50.00
	N7	272	152	35	1.46%	427	2.80	130	45.00
	N9	272	152	31	1.46%	427	2.80	85	42.00
	N11	272	152	33	0.97%	427	2.80	75	37.00
	N12	272	152	28	1.46%	628	5.61	30	48.00
	N13	272	152	31	1.46%	628	5.61	40	50.00
	N14	272	152	31	1.46%	427	2.80	40	50.00
	N15	272	152	32	1.46%	427	2.80	20	50.00
	N16	272	152	31	1.46%	628	1.96	40	52.00
	N18	272	152	31	1.46%	427	2.80	60	45.00
	N19	272	152	29	1.46%	427	2.80	80	40.00
	N20	272	152	46	1.46%	427	2.80	60	42.00
	N21	272	152	15	1.46%	427	2.80	60	40.00
	N22	272	152	32	1.46%	427	1.96	60	85.00
	N23	272	152	35	1.46%	427	1.96	20	75.00
	N24	272	152	22	1.46%	427	2.80	60	37.00
^40^	M5	250	760	21	0.4%	602	4.00	295	137.30
	M6	250	760	27	0.5%	643	4.00	393	137.30
^41^	T4	262	200	53	1.8%	534	2.50	327	94.00
	T5	262	200	53	1.8%	534	2.50	439	81.90
	T6	262	200	53	1.8%	534	2.50	223	126.50
^42^	PB4	890	70	16	1.1%	423	N/A	72	72.30
	PB6	890	70	17	1.1%	425	N/A	72	71.60
	PB7	890	70	20	1.1%	425	N/A	102	53.60
	PB8	890	70	20	1.1%	425	N/A	148	49.20
	PB10	890	70	24	1.1%	425	N/A	148	34.90
	PB16	890	70	42	1.1%	502	N/A	181	90.30
	PB14	890	70	42	2.0%	489	N/A	288	95.90
	PB17	890	70	25	2.0%	502	N/A	449	76.00
	PB19	890	70	20	2.0%	402	N/A	80	79.70
	PB20	890	70	22	2.0%	411	N/A	177	88.50
	PB28	890	70	23	2.0%	424	N/A	191	95.30
	PB21	890	70	22	2.0%	426	N/A	274	88.50
	PB22	890	70	18	2.0%	402	N/A	392	64.20
	PB29	890	70	42	2.0%	433	N/A	186	92.80
	PB30	890	70	40	2.0%	496	N/A	277	92.20
	PB31	890	70	43	2.0%	496	N/A	422	71.60
^43^	ZS2	164	600	40	4.0%	500	3.05	1200	356.00
^44^	P1	178	600	35	0.2%	477	3.82	60	92.00
	P2	178	600	35	0.2%	477	3.82	60	92.00
	P3	178	600	43	0.2%	477	3.82	60	92.00
	P4	178	600	43	0.6%	506	3.82	60	92.00
	P5	178	600	35	0.6%	506	3.82	60	92.00
^45^	ST9	278	290	46	1.95%	536	3.60	280	69.90
	ST10	278	290	46	1.95%	536	3.60	525	65.60
	ST11	278	290	46	1.95%	536	3.60	776	65.60
	ST12	278	290	46	1.95%	536	3.60	1507	47.10
	ST13	278	290	46	1.95%	536	3.60	1050	65.60
	ST25	278	290	59	1.00%	484	3.60	165	82.00
	ST26	278	290	59	1.00%	484	3.60	191	58.90
^46^	S1	204	80	37	0.87%	356.5	2.00	30	32.75
	S2	204	80	37	0.87%	356.5	2.00	50	28.85
	S3	204	80	37	0.87%	356.5	2.00	60	27.95
	S4	204	80	37	0.87%	356.5	2.00	70	23.85
	S5	204	80	37	0.87%	356.5	2.50	30	27.00
	S6	204	80	37	0.87%	356.5	2.50	50	23.80
	S7	204	80	37	0.87%	356.5	2.50	60	23.35
	S8	204	80	37	0.87%	356.5	2.50	70	22.30
	S10	204	100	37	1.26%	356.5	2.00	20	30.03
	S11	204	100	37	1.26%	356.5	2.00	30	24.34
	S12	204	100	37	1.26%	356.5	2.00	40	23.00
	S14	204	100	37	1.26%	356.5	2.50	20	24.15
	S15	204	100	37	1.26%	356.5	2.50	30	19.42
	S16	204	100	37	1.26%	356.5	2.50	40	15.43
^30^	A1	250	400	47	1.6%	579	3.00	450	150.00
	A2	250	400	47	1.6%	579	3.00	340	146.60
	A2′	250	400	47	1.6%	579	5.00	340	122.90
	A3	250	400	49	1.6%	579	3.00	560	119.80
	A3′	250	400	49	1.6%	579	5.00	560	124.50
	A4	250	400	49	2.5%	559	3.00	340	162.50
	A4′	250	400	49	2.5%	559	5.00	340	134.10
	A5	250	400	49	1.0%	585	3.00	340	146.30
	B2	250	400	46	2.0%	558	3.00	200	122.50
	B3	250	400	46	2.0%	558	3.00	400	164.40
	B3′	250	400	46	2.0%	558	5.00	400	132.70
	B4	250	400	46	2.0%	558	3.00	600	109.80
	B4′	250	400	46	2.0%	558	5.00	600	125.20
	B5	250	400	48	2.0%	558	3.00	800	139.40
	B5′	250	400	48	2.0%	558	5.00	800	113.30
	B6	250	400	46	1.0%	572	3.00	200	137.30
	B7	250	400	44	1.6%	546	3.00	200	144.60
	B7′	250	400	44	1.6%	546	5.00	200	109.00
	B8	250	400	45	2.5%	570	3.00	200	150.20
	B9	250	400	45	2.8%	566	3.00	200	150.80
	B9′	250	400	45	2.8%	566	5.00	200	143.60
	B10	250	400	48	1.0%	572	3.00	600	94.10
	B11	250	400	47	1.6%	546	3.00	600	160.10
	B11′	250	400	47	1.6%	546	5.00	600	126.30
	B12	250	400	47	2.5%	570	3.00	600	174.00
	B12′	250	400	47	2.5%	570	5.00	600	140.70
	C1	250	400	43	1.6%	559	3.00	200	249.60
	C1′	250	400	43	1.6%	559	5.00	200	149.70
	C2	250	400	43	1.6%	559	3.00	600	136.10
	C2′	250	400	43	1.6%	559	5.00	600	153.20
	C4	250	400	44	1.6%	559	4.00	150	144.20
	C4′	250	400	44	1.6%	559	4.00	150	136.10
	C5	250	400	44	1.6%	559	4.00	340	138.50
	C7	250	400	44	1.6%	559	4.00	150	129.80
	C7′	250	400	44	1.6%	559	4.00	150	125.10
	C8	250	400	45	1.6%	559	4.00	340	127.60
	C8′	250	400	45	1.6%	559	4.00	340	116.70
	C9	250	400	27	2.0%	554	4.00	500	105.20
	C10	250	400	52	2.0%	554	3.00	500	146.00
	C11	250	400	45	1.5%	550	3.00	340	146.40
	C12	250	400	45	1.5%	550	3.00	600	151.20
	C12′	250	400	45	1.5%	550	5.00	600	143.00
	C13	250	400	46	2.0%	554	3.00	900	111.20
	C13′	250	400	46	2.0%	554	5.00	900	134.50
^47^	ST-1	165	200	25	1.1%	1027	2.25	427	39.50
	ST-2	165	200	26	1.1%	1027	2.25	97	43.50
	ST-3	165	200	26	1.1%	1027	2.25	200	45.40
	ST-6	165	200	27	1.1%	1027	2.25	300	43.00
	ST-7	165	200	27	1.1%	1027	2.25	401	40.80
	ST-8	165	200	27	1.1%	1027	2.25	499	39.80
	ST-9	165	200	27	1.1%	1027	2.25	299	45.40
	ST-10	165	200	28	1.1%	1027	2.25	401	36.90
	ST-12	165	200	28	1.1%	1027	2.25	200	44.00
	ST-13	165	200	29	1.1%	1027	2.25	100	33.90
	ST-14	165	200	29	1.1%	1027	2.75	201	40.70
	ST-15	165	200	29	1.1%	1027	2.75	301	44.60
	ST-17	165	200	30	1.1%	1027	2.75	100	39.90
	ST-18	165	200	30	1.1%	1027	2.75	200	32.80
	ST-19	165	200	30	1.1%	1027	2.75	300	32.00
	ST-20	165	200	30	1.1%	1027	2.75	500	30.20
	ST-22	165	200	30	1.1%	1027	2.75	501	37.50
	ST-23	165	200	30	1.1%	1027	2.25	602	34.50
	ST-24	165	200	30	1.1%	1027	2.25	600	35.20
^48^	V8-1	164	140	36	1.00%	495	1.97	30	57.37
	V8-2	164	140	82	1.00%	495	1.97	51	75.73
	V8-3	164	140	34	1.00%	495	1.97	50	45.13
	V8-4	164	140	34	1.00%	495	1.97	102	50.91
	V9-1	164	140	31	1.51%	487	1.97	27	68.94
	V9-2	164	140	74	1.51%	487	1.97	47	71.95
	V9-3	164	140	36	1.51%	487	1.97	60	52.83
	V9-4	164	140	74	1.51%	495	1.97	69	109.90
	V9-5	164	140	33	1.51%	495	1.97	109	58.09
	V9-6	164	140	82	1.51%	487	1.97	154	52.63
^33^	ST1	267	4000	34	1.15%	500	4.59	600	711.00
	ST2	267	4000	35	1.15%	500	4.59	780	742.00
	ST3	267	4000	34	1.15%	500	4.59	1200	539.00
	ST4	267	4000	34	1.15%	500	4.59	1440	555.00
^49^	SC8	267	4000	35	1.15%	500	4.59	1200	801.00
	SC9	267	4000	33	1.15%	500	4.59	1800	792.00
^32^	N1-1	255	300	38	1.00%	957	4.53	259	73.00
	N1-2	255	300	39	1.00%	957	4.53	258	70.00
	N2-1	255	300	38	1.00%	957	4.53	195	102.00
	N2-2	255	300	39	1.00%	957	4.53	195	55.00
	N3-1	255	300	38	1.00%	957	4.53	317	68.00
	N3-2	255	300	39	1.00%	957	4.53	317	116.00
^31^	4	280	200	33	1.65%	550	3.57	147	68.00
	5	280	200	33	1.65%	550	3.57	147	51.00
	6	280	200	35	1.65%	550	3.57	148	59.00
	7	280	200	33	1.65%	550	3.57	298	60.00
	8	280	200	33	1.65%	550	3.57	297	48.00
	9	280	200	35	1.65%	550	3.57	297	56.00
	10	280	200	34	1.65%	550	3.57	397	43.00
	11	280	200	34	1.65%	550	3.57	397	61.00
	12	280	200	35	1.65%	550	3.57	397	62.00
	13	280	200	34	1.65%	550	3.57	596	63.00
	14	280	200	34	1.65%	550	3.57	596	60.00
	15	280	200	35	1.65%	550	3.57	594	73.00
Average		296	370	37	1.5%	563	3.35	292	100.05
Minimum		164	70	15	0.2%	343	1.96	20	15.43
Maximum		890	4000	82	4.0%	1027	5.61	1800	801

## Model development

### Introduction

**Figure 4:**
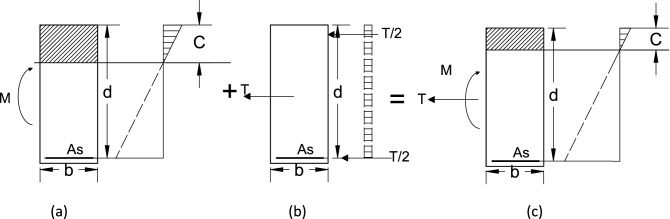
Strain diagram for (**a**) flexure only, (**b**) tension only, and (**c**) flexure and tension.

This work is inspired by the Critical Shear Crack Theory (CSCT)^28^, which was first introduced in the 1990s and later implemented in the swiss design code, MC, and the new draft of the Eurocode^26,50^. The shear strength (${v}_{u}$) is calculated such that:

1 $$\frac{{v}_{u}}{\sqrt{{f}_{c}^{\prime}}}=\text{f}(\omega ,{\text{d}}_{dg})$$

while $\upomega$ is the crack width, ${f}_{c}^{\prime}$ is the cylinder compressive strength and ${d}_{dg}$ is the maximum nominal aggregate size.

2 $$\omega \propto \varepsilon d$$

where $d$ is the effective depth, and $\varepsilon$ is the longitudinal strain, which is taken at 60% of the effective depth from extreme compression fibers^28^. In addition, the following assumptions were implemented, which are similar to the work by Deifalla^24,25^ for slabs under combined punching shear and tension: (1) Plane cross-sections before deformation remain plain after deformation,m while maintaining small deformation. (2) Concrete in compression is linear elastic behavior. (3) Concrete in tension is neglected. (4) Steel reinforcements reached yield. (5) The superposition principle applies to the longitudinal strains from flexure and tension. Figure 4a–c shows the distribution diagrams for a) flexure only, b) tensile forces only, and c) both, respectively; thus, the tensile strain at 60% of the effective depth from extreme compression fibers $\left(\upvarepsilon \right)$, which is calculated such that:

3 $$\varepsilon =\frac{1}{bd{\varvec{\rho}}{E}_{s}}\left[\frac{M}{d\left(1-{\varvec{\rho}}\cdot \frac{{E}_{s}}{{E}_{c}}\left(\sqrt{1+\frac{2{E}_{c}}{{\varvec{\rho}}\cdot {E}_{s}}}-1\right)/3\right)}+\frac{N}{2}\right]\left(\frac{0.6d-c}{d-c}\right)$$

where $b$ is the element width, ${\varvec{\rho}}$ is the flexure reinforcement ratio, $N$ is the axial force ( positive is tension and negative is compression), $M$ is the bending moment at the critical section for shear, ${E}_{s}$ is the steel reinforcements young’s modulus (210,000 MPa), ${E}_{c}$ is the concrete young’s modulus (10,000$\sqrt[3]{{f}_{c}^{^{\prime}}}$), $c$ is the compression zone depth, which is calculated such that:

4 $$\frac{c}{d}={\varvec{\rho}}\cdot \frac{{E}_{s}}{{E}_{c}}\left(\sqrt{1+\frac{2{E}_{c}}{{\varvec{\rho}}\cdot {E}_{s}}}-1\right)-\frac{Nd/M}{\left[\frac{2}{\left(1-{\varvec{\rho}}\cdot \frac{{E}_{s}}{{E}_{c}}\left(\sqrt{1+\frac{2{E}_{c}}{{\varvec{\rho}}\cdot {E}_{s}}}-1\right)/3\right)}+\frac{Td}{M}\right]}$$

**Figure 5:**
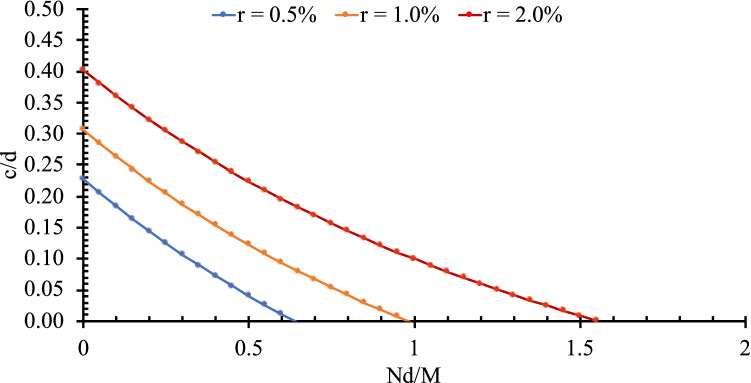
The effect of tension forces on the compression zone depth of elements with different reinforcement ratios (*f*c′ = 30 MPa and *M*/*Vd* = 4).

Figure 5 shows the variation of the compression zone depth (Eq. 4) versus the tensile stress for different flexure reinforcement ratios. Figure 5 was based on ${f}_{c}^{^{\prime}}$ value of 30 MPa and *M*/*Vd* value of 4, where V is the shear force at the critical section for shear. It is clear that the model captured the reduction in the compression zone depth observed by Pham^31^.

### Proposed failure criteria for combined shear and tension

Based on data shown in Table 1 and Fig. 3 as well as the failure criteria of the CSCT, the following form was proposed:

5 $$\frac{{v}_{u}}{\sqrt{{f}_{c}^{\prime}}}=\frac{{\alpha }_{1}}{1+{\alpha }_{2}\frac{\varepsilon d}{{d}_{dg}}}$$

Using nonlinear multi-variable regression, thus, the shear stress is calculated such that:

6 $$\frac{{v}_{u}}{\sqrt{{f}_{c}^{\prime}}}=\frac{0.27}{1+22\frac{\varepsilon d}{{d}_{dg}}}$$

for shear and tension. Where ${v}_{u}=\frac{V}{b d}$, ${d}_{dg}={d}_{g0}+{d}_{g}$ (the value of the reference aggregate size ${d}_{g0}=16$ mm is used), and ${d}_{g}$ is the maximum nominal aggregate size. Table 2 shows the parameter table for the coefficient ${\alpha }_{1}$ and ${\alpha }_{2}$.

**Table 2:** Parameter table for ECSCT.

Parameter	Estimate	Standard error	t-Statistic	p-value
${\alpha }_{1}$	0.27	0.019	13.7	7.79E − 30
${\alpha }_{2}$	22	8.47	2.56	0.011

## Simplified model

### Model development

In this section, a simplified model is developed. Based on the proposed mechanical model presented in Eqs. (3), (4) and (6), the following parameters are identified to be effective in the shear strength of elements subjected to axial tensile forces: (1) $M/Nd$, (2) $N/(bd{\varvec{\rho}}{f}_{y})$, (3) $M/Vd$ or $a/d$, (4) $d$, (5) ${f}_{c}^{\prime}$, and (6) $\rho$. Thus, nonlinear multi-variable regression was performed using the following power form:

7 $${v}_{u}={{\varvec{c}}}_{1}{\left(N/(bd{\varvec{\rho}}{f}_{y})\right)}^{{{\varvec{c}}}_{2}}{{\left(M/Nd\right)}^{{{\varvec{c}}}_{3}}{{\varvec{\rho}}}^{{{\varvec{c}}}_{4}}{f}_{c}^{^{\prime}}}^{{{\varvec{c}}}_{5}}{\left(M/Vd\right)}^{{{\varvec{c}}}_{6}}{d}^{{{\varvec{c}}}_{7}}$$

Power form was implemented in several investigations (Ali et al., 2021; Deifalla et al., 2021; Deifalla, 2020b; 2020c; 2021b; 2021c). Table 3 shows the parameter table, including the probability of each parameter. It is worth noting that the power coefficient of the variable $N/(bd{\varvec{\rho}}{f}_{y})$ failed the hypotheses test, and it was found to be insignificant. Therefore, it is proposed that shear strength is calculated such that:

**Table 3:** Parameter table for simplified model.

Parameter	Estimate	Standard error	t-statistic	p-value	Lower 95%	Upper 95%
${c}_{1}$	17.22	0.530	5.37	2.53E − 07	6.047	49.05
${c}_{2}$	0.010	0.042	0.24	0.81	− 0.074	0.094
${c}_{3}$	0.077	0.017	4.41	1.81E − 05	0.042	0.111
${c}_{4}$	0.34	0.042	8.00	1.65E − 13	0.258	0.427
${c}_{5}$	0.40	0.063	6.29	2.39E − 09	0.273	0.523
${c}_{6}$	− 0.70	0.064	− 11	1.56E − 21	− 0.83	− 0.576
${c}_{7}$	− 0.39	0.054	− 7.1	2.31E − 11	− 0.495	− 0.281

8 $${v}_{u}=17.22{{\left(\text{M}/\text{Nd}\right)}^{0.077}{{\varvec{\rho}}}^{0.34}{f}_{c}^{\prime}}^{0.4}{\left(M/Vd\right)}^{-0.7}{d}^{-0.39}$$

## Models validation

For simplicity, two design codes were selected for comparison: the ACI and the EC2. However, it is worth noting that there is other design model that are more accurate with various levels of approximation, for example fib model code^50^. For simplicity, the model code was not selected as it requires a detailed calculation compared to the ACI and EC2. The strength calculated using the ECSCT and the simplified model were assessed against those calculated using the selected design codes with respect to the experimentally measured strength.

Several types of figures were implemented to compare the performance of the proposed models with the selected design codes, which were implemented in several investigations^12,51–54^. Firstly: a scatter plot between the measured and calculated strength was plotted for all models, which was assessed using the ideal 45-degree line and the inverse of the slope of the best-fitted line. While the strength in terms of stress is taken as the ratio between the shear force and the concrete cross area. Secondly: a histogram figure for the distribution of the ratio between measured and calculated strength (SR), which is assessed based on the distribution and being far from the ideal ratio of unity and lower coefficient of variation. The unity value for SR indicates the closeness of the calculated value to the measured one (i.e., the model accuracy). At the same time, the coefficient of variation of the SR distribution indicates the consistency of the model. In addition, the lower 95% of the SR indicates the safety of the model. It is the minimum SR value obtained using the model with a 95% confidence level. Therefore, the higher value of the lower 95% limit above the safety factor of design codes (approximately 0.85), the safer the model is. The confidence interval is calculated assuming a standard normal distribution. In addition, a significant level value of 0.05 represents the 95% confidence level. Thus, the lower 95% confidence limit is calculated using the following expression:

8 $${\text{Lower}}\;95\text{\%}={\text{Average}}-1.96\left(\frac{\text{Standard deviation}}{\sqrt{\text{number of samples}}}\right)$$

Thirdly: a scatter plot for the SR against various effective parameters is plotted, which is assessed using the inclination of the best-fitted line and the correlation coefficient (*r*). The closer the slope to zero is the more negligible effect of the variable on the accuracy and safety of the model. The *r* is the degree of association, which is such that:

$$r=\left|\frac{\sum_{i=1}^{n}\left({x}_{i}-\overline{x }\right)\left({y}_{i}-\overline{y }\right)}{\sqrt{\sum_{i=1}^{n}{\left({x}_{i}-\overline{x }\right)}^{2}}\sqrt{\sum_{i=1}^{n}{\left({y}_{i}-\overline{y }\right)}^{2}}}\right| (9)$$

where, $\overline{x }$ and $\overline{y }$ are average values of variables ${x}_{i}$ and ${y}_{i}$, respectively, and n is the number of tested specimens. The correlation coefficient is measured on a scale that varies from $\pm 1$ to 0. For example, $\pm 1$, $\pm 0.70$, $\pm 0.50$, $\pm 0.30$, and zero indicate exact, strong, moderate, weak, and no dependence, respectively. Thus, if the r value is less than $\pm 0.30$ shows that the model captured the effect of such parameter, while a coefficient ranged between $\pm 0.50$ and $\pm 0.30$ indicates a need for refinements in the modeling of this parameter. It is worth noting that the correlation coefficient is not directly related to the data scattering. Because the data scattering is dependent on the overall effect of the considered parameters in the specific model, while the correlation coefficient is indicative of the relation between the accuracy and a specific parameter. It is an indication not conclusive depending on the value as mentioned before.

### Overall

**Figure 6:**
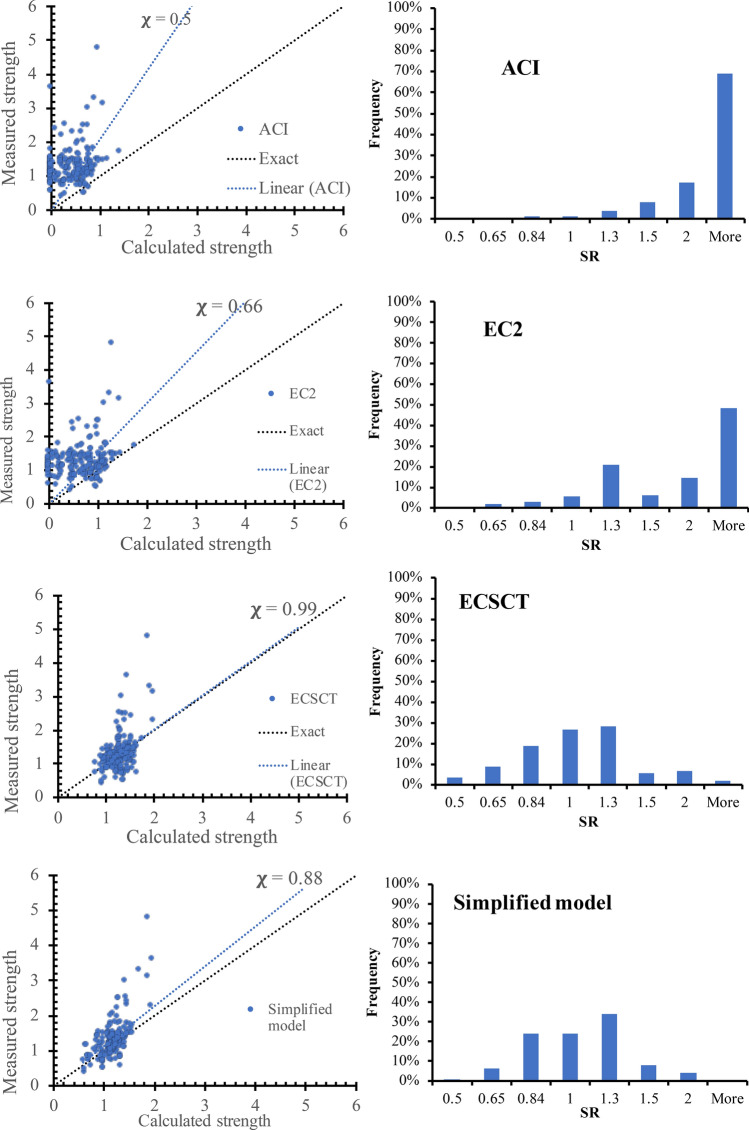
Measured Strength versus calculate strength using various methods.

Figure 6 shows the calculated shear strength versus the measured ones for the ECSCT, the simplified model, the ACI, and the EC2. While the strength is calculated in terms of stress taken as the ratio between the shear force and the concrete cross area. In addition, the line represents the actual performance and the linear fitted line for the model performance. Moreover, the inverse of the best-fitted line slope ($\chi$) is indicated in the plots. The closer this value to unity is, the better accuracy and less divergence. The $\chi$ value for the ECSCT, the simplified model, the ACI, and the EC2 is 0.99, 0.88, 0.50, and 0.65. Thus, the strength calculated using the ECSCT and the simplified model is significantly less scattered than using the ACI and EC2.

Moreover, Fig. 6 shows the histogram of the SR values calculated using the ECSCT, the simplified model, the ACI, and the EC2. The SR is calculated using the simplified model, and the ECSCT is normally distributed around the ratio of unity compared to that using the ACI and the EC2. Last but not least, Table 4 shows the average, coefficient of variation, and lower 95% limit for SR calculated using the ECSCT, the simplified model, the ACI, and the EC2 for each study. It is clear that the performance of the ECSCT and the simplified model is more accurate and consistent than that of the ACI and EC2. However, it is safe with a lower 95% value of 0.96, higher than 0.85 targeted by most design codes. The simplified model and ECSCT SR values have a narrow range compared to that calculated using the ACI and the EC2, as shown in Table 4.

**Table 4:** Statistical measures for the SR for shear with axial tension.

Refs.	Measure	Number	ACI-19	EC2	ECSCT	Simplified model
^36^	Mean	2	1.80	0.95	0.48	0.74
	Coefficient of variation		2%	6%	14%	12%
^37^	Mean	11	1.49	1.03	0.93	1.04
	Coefficient of variation		22%	17%	16%	20%
^38^	Mean	3	2.11	1.40	1.25	0.95
	Coefficient of variation		19%	19%	19%	24%
^39^	Mean	19	1.86	1.23	0.92	0.87
	Coefficient of variation		23%	21%	24%	22%
^40^	Mean	2	3.00	1.58	0.85	1.19
	Coefficient of variation		10%	7%	13%	12%
^41^	Mean	3	5.00	5.00	1.27	1.04
	Coefficient of variation		0%	0%	21%	18%
^42^	Mean	16	5.00	4.62	1.16	1.23
	Coefficient of variation		0%	13%	21%	21%
^43^	Mean	1	5.00	5.00	2.50	1.71
	Coefficient of variation		N/A	N/A	N/A	N/A
^44^	Mean	5	2.84	1.53	0.73	1.02
	Coefficient of variation		23%	20%	15%	19%
^45^	Mean	14	3.17	1.89	1.06	0.90
	Coefficient of variation		48%	43%	28%	27%
^46^	Mean	7	3.25	2.75	0.56	0.64
	Coefficient of variation		59%	77%	13%	14%
^30^	Mean	44	3.16	2.23	0.95	1.02
	Coefficient of variation		43%	50%	18%	18%
^47^	Mean	19	4.73	4.46	1.11	0.93
	Coefficient of variation		33%	27%	14%	11%
^48^	Mean	10	4.19	3.00	1.81	1.25
	Coefficient of variation		18%	27%	22%	17%
^33^	Mean	4	0.83	0.59	0.44	0.62
	Coefficient of variation		10%	11%	16%	12%
^49^	Mean	2	1.16	0.81	0.58	0.80
	Coefficient of variation		11%	16%	3%	3%
^32^	Mean	6	2.80	1.70	0.88	1.15
	Coefficient of variation		44%	38%	34%	27%
^31^	Mean	12	4.24	3.22	0.88	0.93
	Coefficient of variation		32%	53%	18%	14%
Overall	Mean	180	3.3	2.5	1.01	0.99
	Coefficient of variation		46%	62%	35%	24%
	Minimum		0.73	0.5	0.37	0.50
	Maximum		5	5	2.58	1.71
	Lower 95.0%		3.07	2.3	0.96	0.96

### Effect of axial tension (*N*)

**Figure 7:**
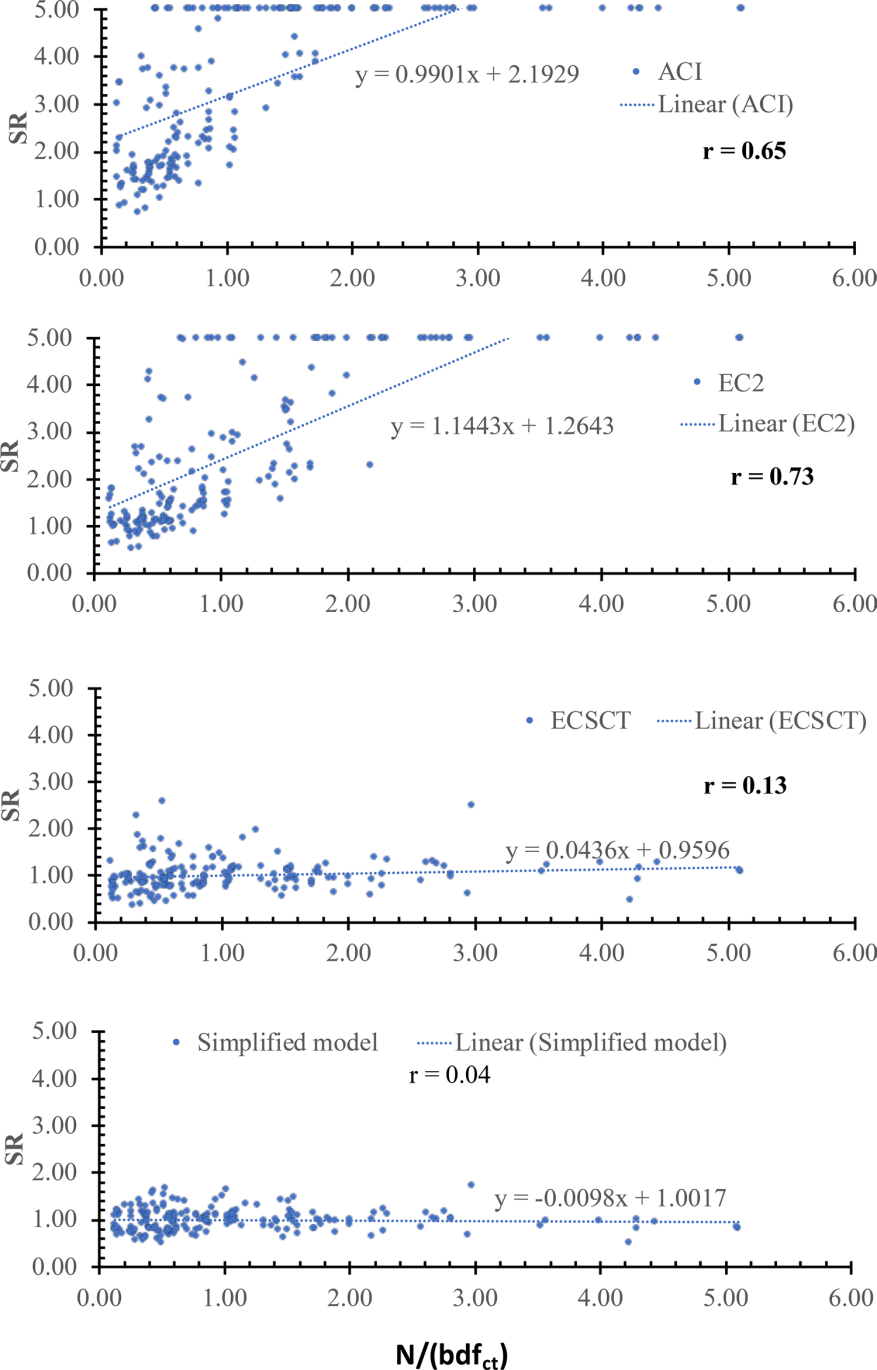
SR using various methods versus the *N*/(*bdf*_*ct*_).

**Figure 8:**
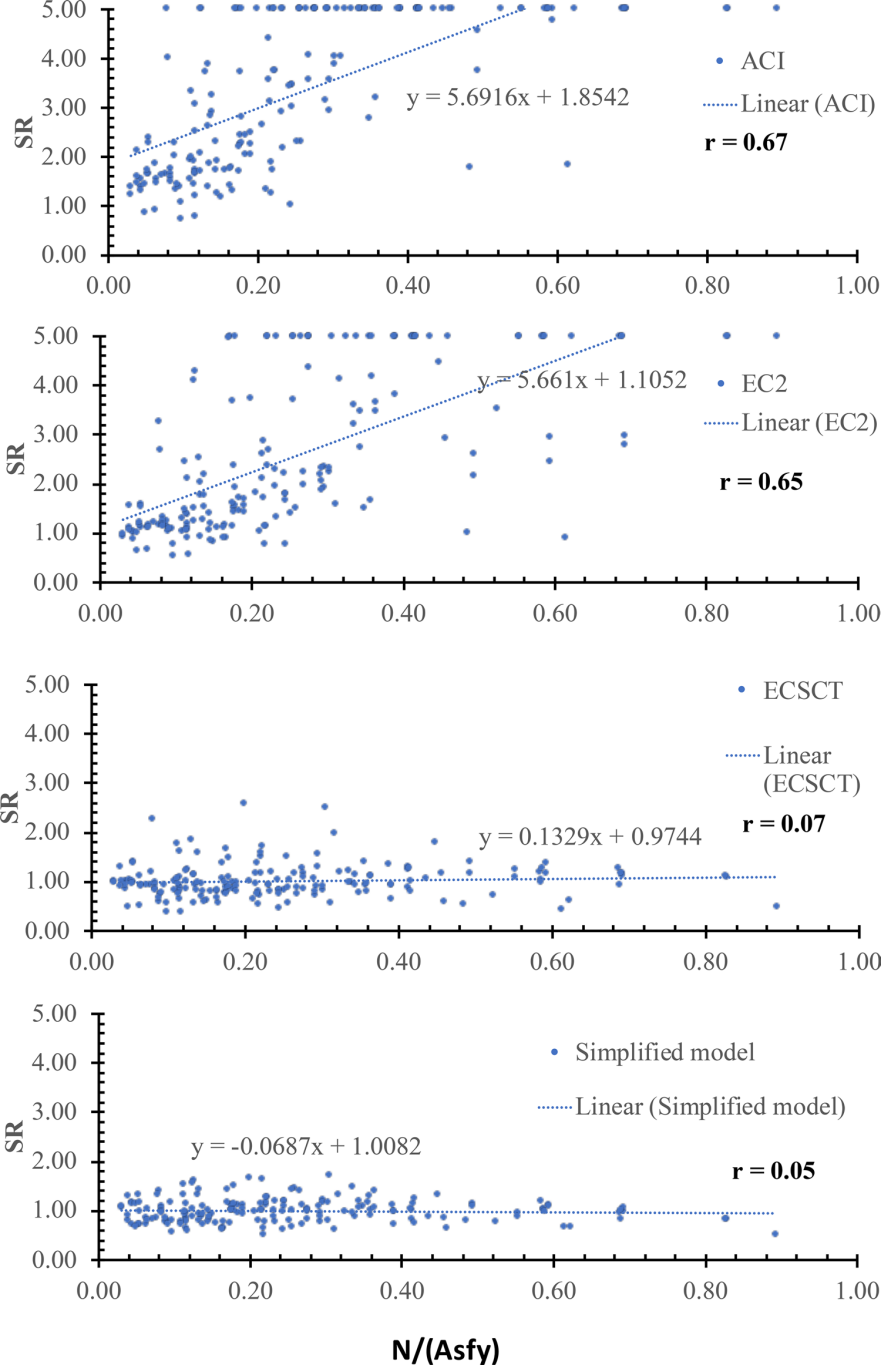
SR using various methods versus the *N*/(***ρbdf***_*y*_).

**Figure 9:**
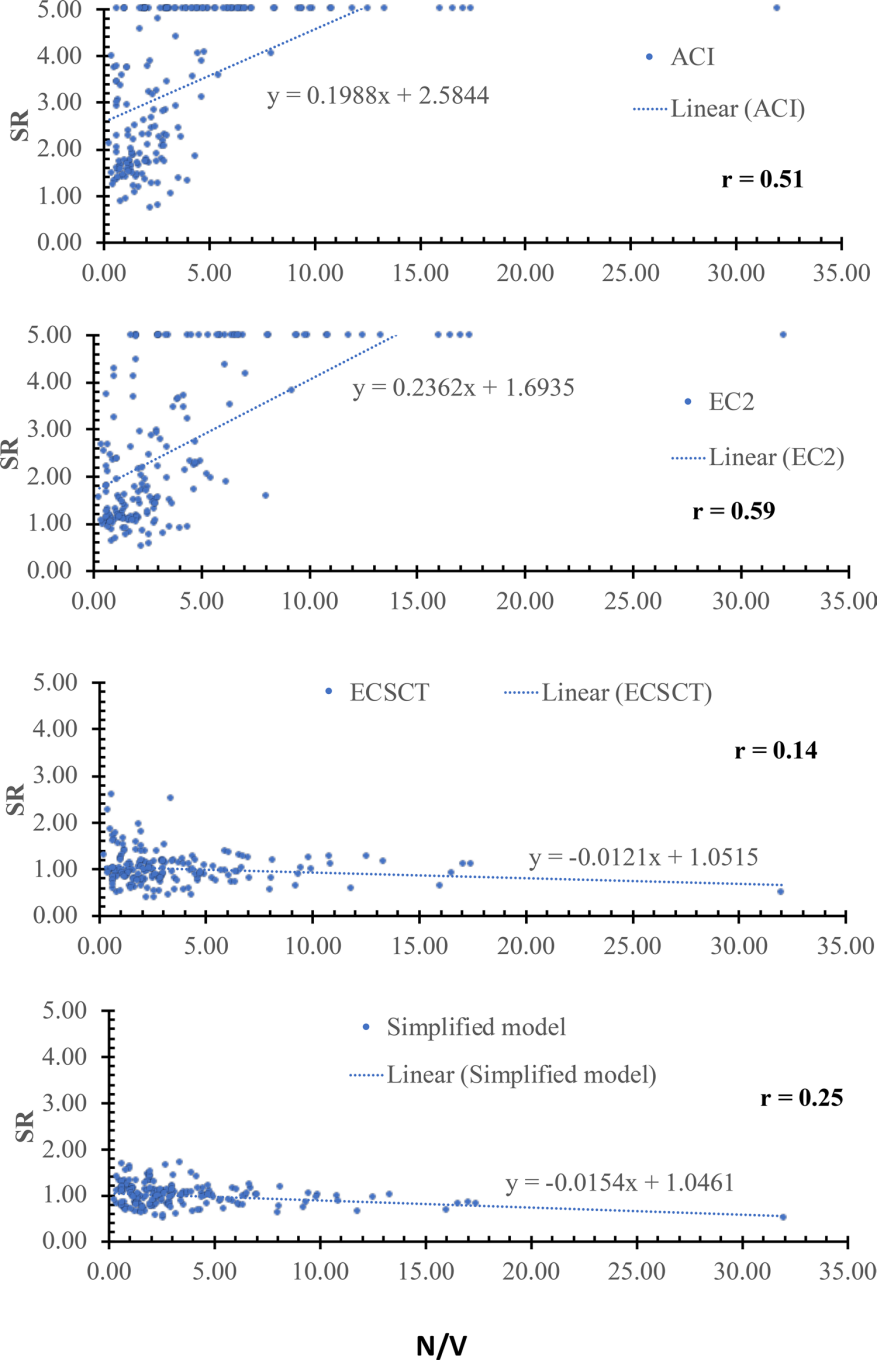
SR using various methods versus the *N*/*V*.

**Figure 10:**
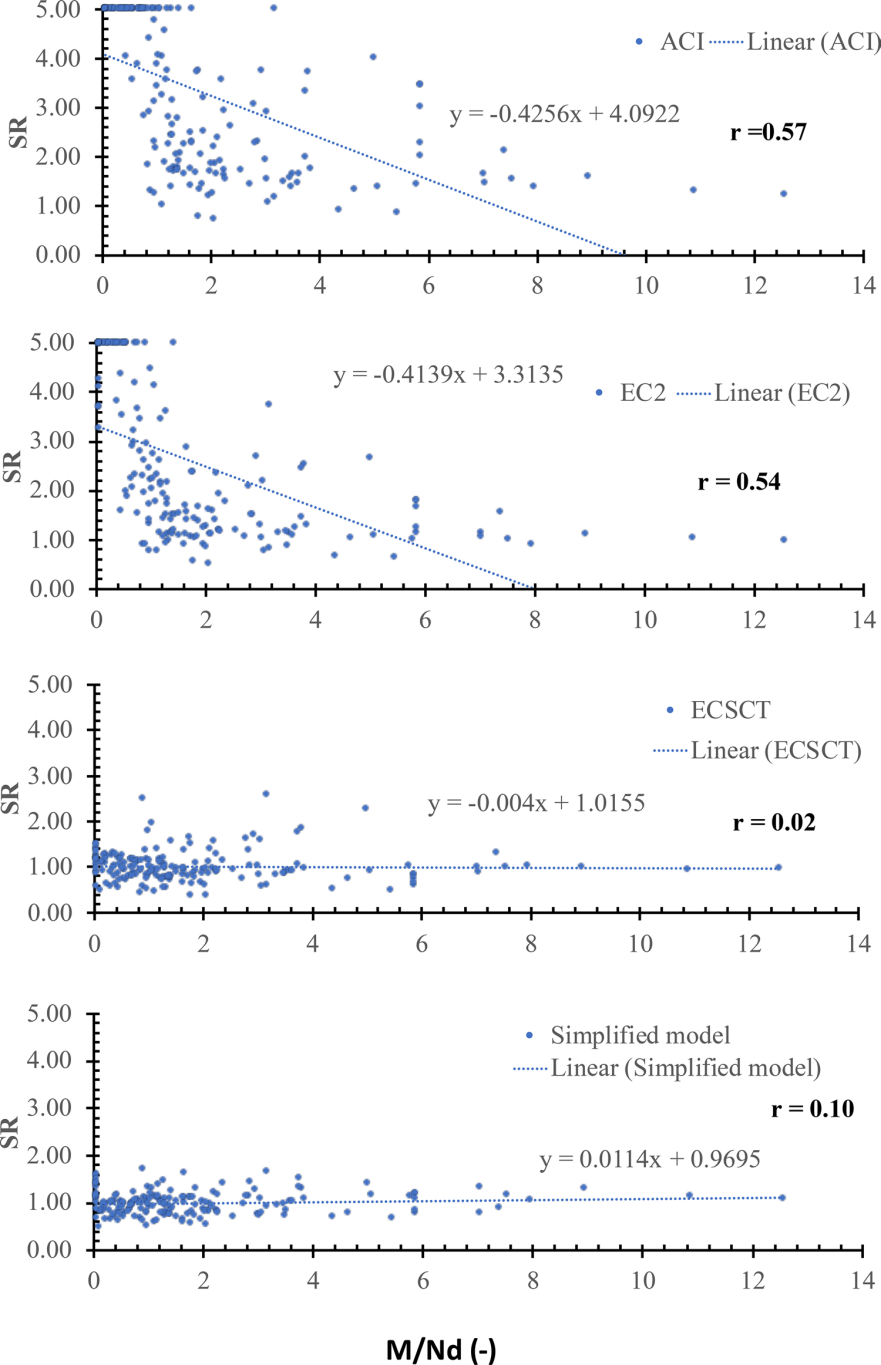
SR using various methods versus the *M*/*Nd.*

The effect of the axial tension was examined using several parameters, namely *N*/(*bdf*_*ct*_), *N*/(***ρbdf***_*y*_), *N*/*V* and *M*/*Nd.* The SR is plotted against the *N*/(*bdf*_*ct*_), *N*/(***ρbdf***_*y*_), *N*/*V* and *M*/*Nd* as shown in Figs. 7, 8, 9 and 10, respectively, where ${f}_{ct}$ is the tensile concrete strength taken as $0.65\sqrt[2]{{f}_{c}^{^{\prime}}}$ , ${f}_{\text{y}}$ is the yield stress of the steel reinforcements. From Figs. 7, 8, 9 and 10, the safety of the strength calculated using the ECSCT and the simplified model is more consistent with the axial tension’s effect than the ACI and EC2. The correlation coefficient (r) was calculated as 0.51–0.67, 0.54–0.73, 0.02–0.14, and 0.04–0.25 for the ACI, the EC2, the ECSCT, the simplified model, respectively. In addition, the slope of the best fit line for the SR calculated using the ACI, the EC2, is much higher than that calculated using the ECSCT, the simplified model. Thus, it is clear that SR calculated using the ECSCT and the simplified model are weakly correlated to the axial tension, while the ACI and EC2 are highly correlated$.$ The variation of the tensile axial force does not affect the ECSCT and the simplified model compared to the ACI and the EC2 with respect to the experimental database.

### Effect of shear span to depth ratio (*a*/*d*)

**Figure 11:**
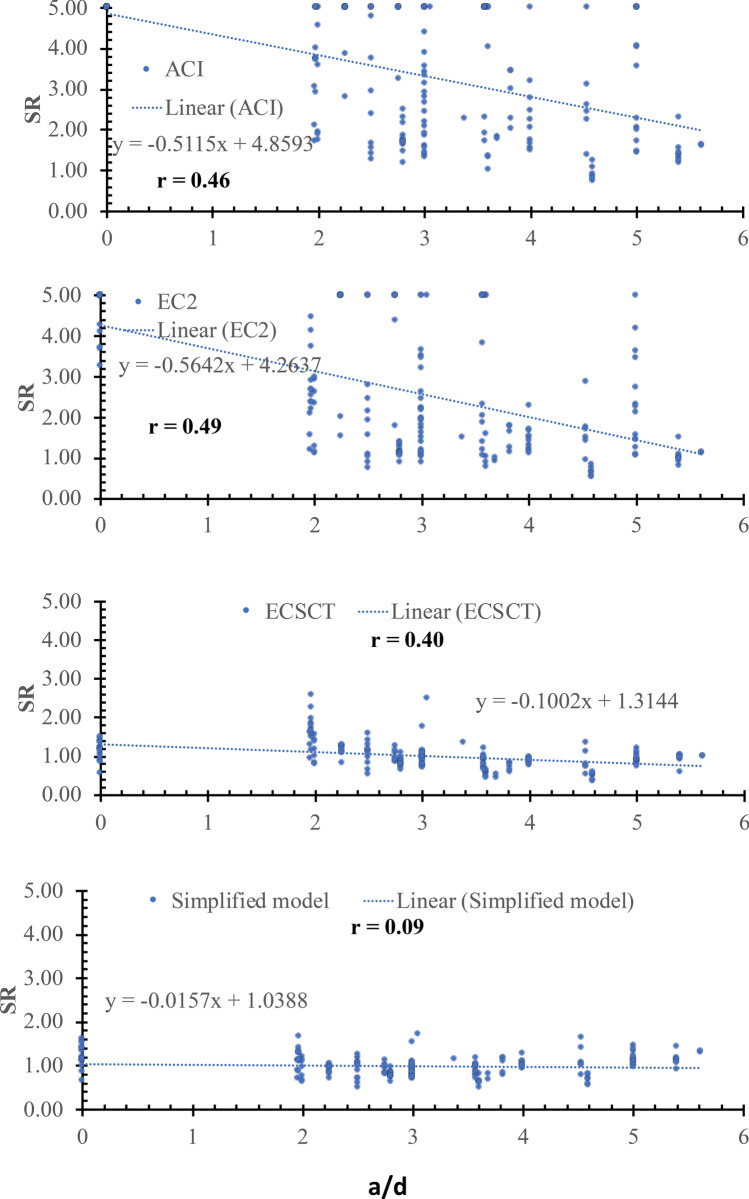
SR using various methods versus the *d*.

The SR is plotted against the specimen size in the shear span to depth ratio (a/d), as shown in Fig. 11. From Fig. 11, the safety of the strength calculated using the ECSCT and the simplified model is more consistent with the effect of the *a*/*d* compared with the ACI and EC2. However, the safety for the ECSCT is higher for non-slender elements with *a*/*d* value less than 3. This is because the original CSCT model was not developed for non-slender. In addition, the correlation coefficient (r) was calculated as 0.46, 0.49, 0.40, and 0.09 for the ACI, the EC2, the ECSCT, the simplified model, respectively. Thus, it is clear that SR calculated using the simplified model is weakly correlated to the *a*/*d*, while the ECSCT, the ACI, and EC2 are highly correlated to the *a*/*d*.

Moreover, the slope of the best fit line for the SR calculated using the ACI, the EC2, the ECSCT, and the simplified model is 0.46, 0.56, 0.10, and 0.02, respectively. The slope for the SR calculated using the ECSCT and simplified model is significantly lower than that calculated using the ACI and the EC2. The simplified model showed quite an improvement with respect to the effect of the arch mechanism in terms of the shear span to depth ratio (a/d).

### Effect of specimen size (*d*)

**Figure 12:**
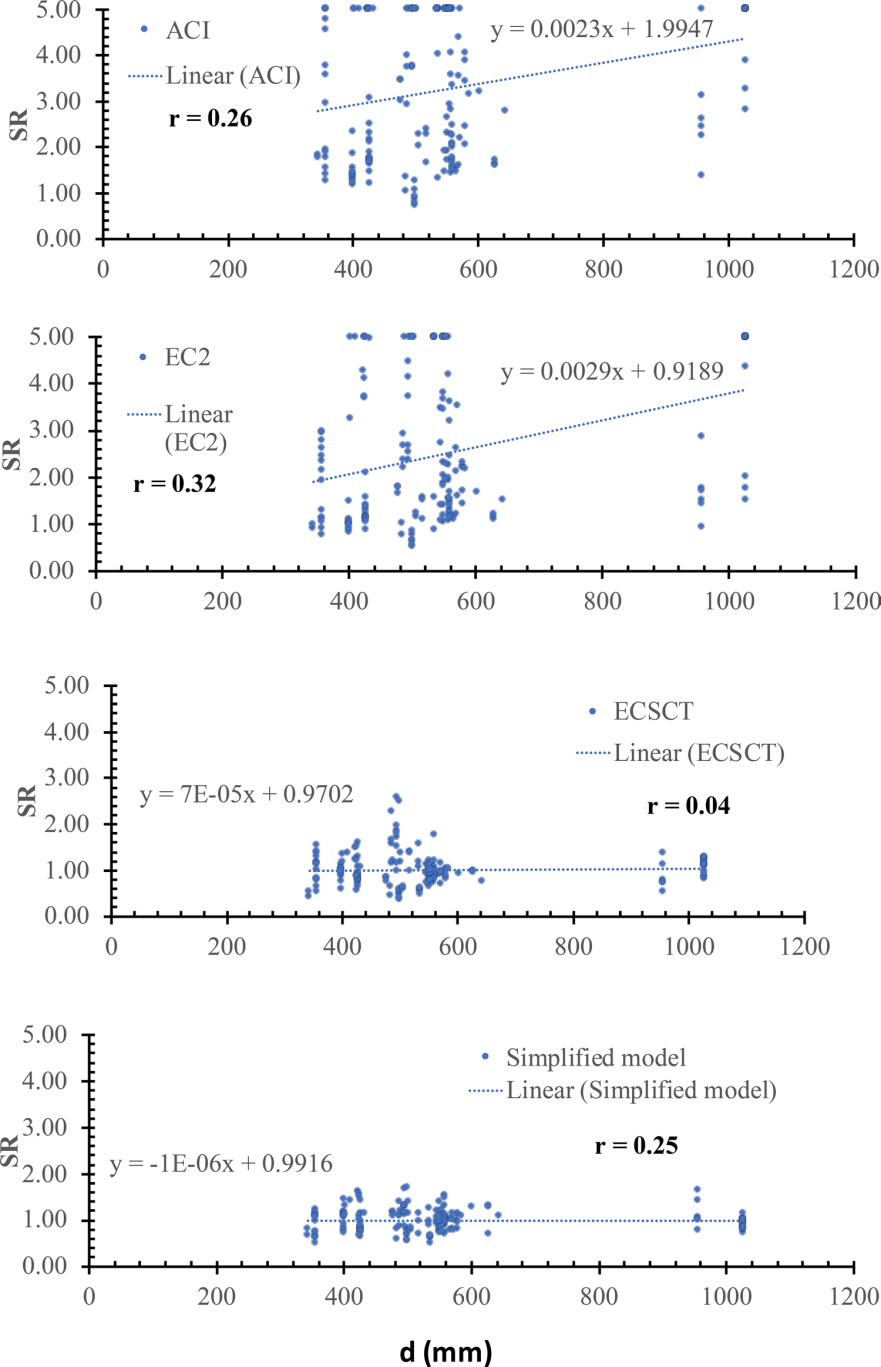
SR using various methods versus the *a*/*d*.

Plots of the SR versus the specimen size in terms of effective depth (*d*) are shown in Fig. 12. From Fig. 12, the safety of the strength calculated using the ECSCT and the simplified model is more consistent with the effect of the *d* compared with the ACI and EC2. In addition, the correlation coefficient (r) was calculated as 0.26, 0.32, 0.04, and 0.25 for the ACI, the EC2, the ECSCT, and the simplified model, respectively. Thus, it is clear that SR calculated using the simplified model, the ECSCT is weakly correlated to the *d*. Moreover, the slope of the best fit line for the SR calculated using the ACI, the EC2, the ECSCT, and the simplified model is 2300E-6, 2900E-6, 70E-6, and 1E-6, respectively. Thus, the slope for the SR calculated using the ECSCT and simplified model is significantly lower than that calculated using the ACI and the EC2. Therefore, the ECSCT model and the simplified model account for the effect of size in terms of *d* much better than the ACI and the EC2.

### Effect of flexural reinforcement ratio (${\varvec{\rho}}$)

**Figure 13:**
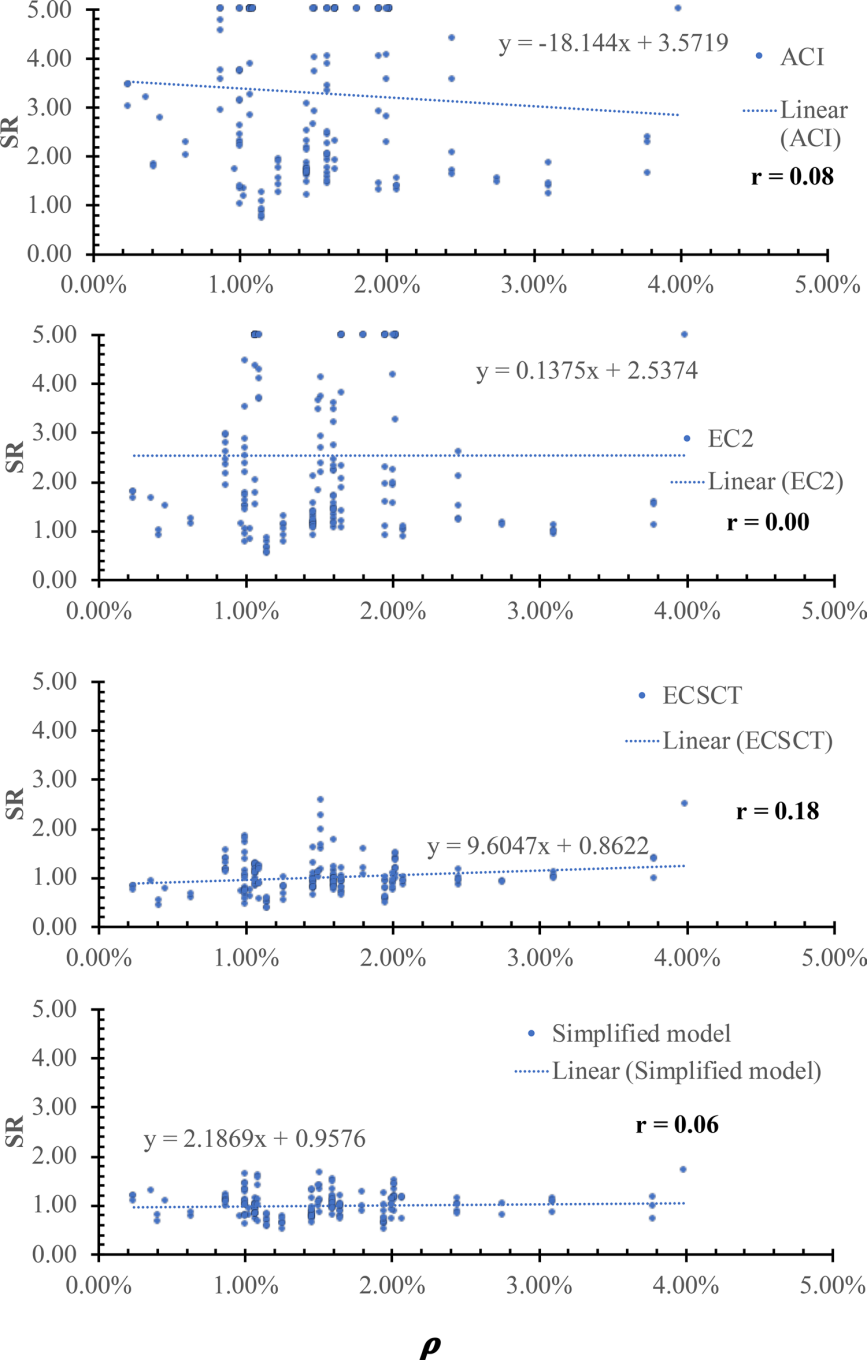
SR using various methods versus the ${\varvec{\rho}}$.

The SR is plotted versus the size in terms of the flexure reinforcement ratio (${\varvec{\rho}}$), as shown in Fig. 13. From Fig. 13, the safety of the strength calculated using the ECSCT and the simplified model is more consistent with the effect of the ${\varvec{\rho}}$ compared with the ACI and EC2. In addition, the correlation coefficient (r) was calculated as 0.08, 0.00, 0.18, and 0.06 for the ACI, the EC2, the ECSCT, and the simplified model, respectively. Thus, it is clear that SR calculated using the ACI, the EC2, the ECSCT, and the simplified model is weakly correlated to the ${\varvec{\rho}}$. Moreover, the slope of the best fit line for the SR calculated using the ACI, the EC2, the ECSCT, and the simplified model is 18.4, 0.137, 9.6, and 2.2, respectively. Thus, the slope for the SR calculated using all models is similar. Therefore, it is clear that all models account for the dowel action mechanism in terms of the flexure reinforcement ratio.

### Effect of concrete compressive strength (*f*c′)

**Figure 14:**
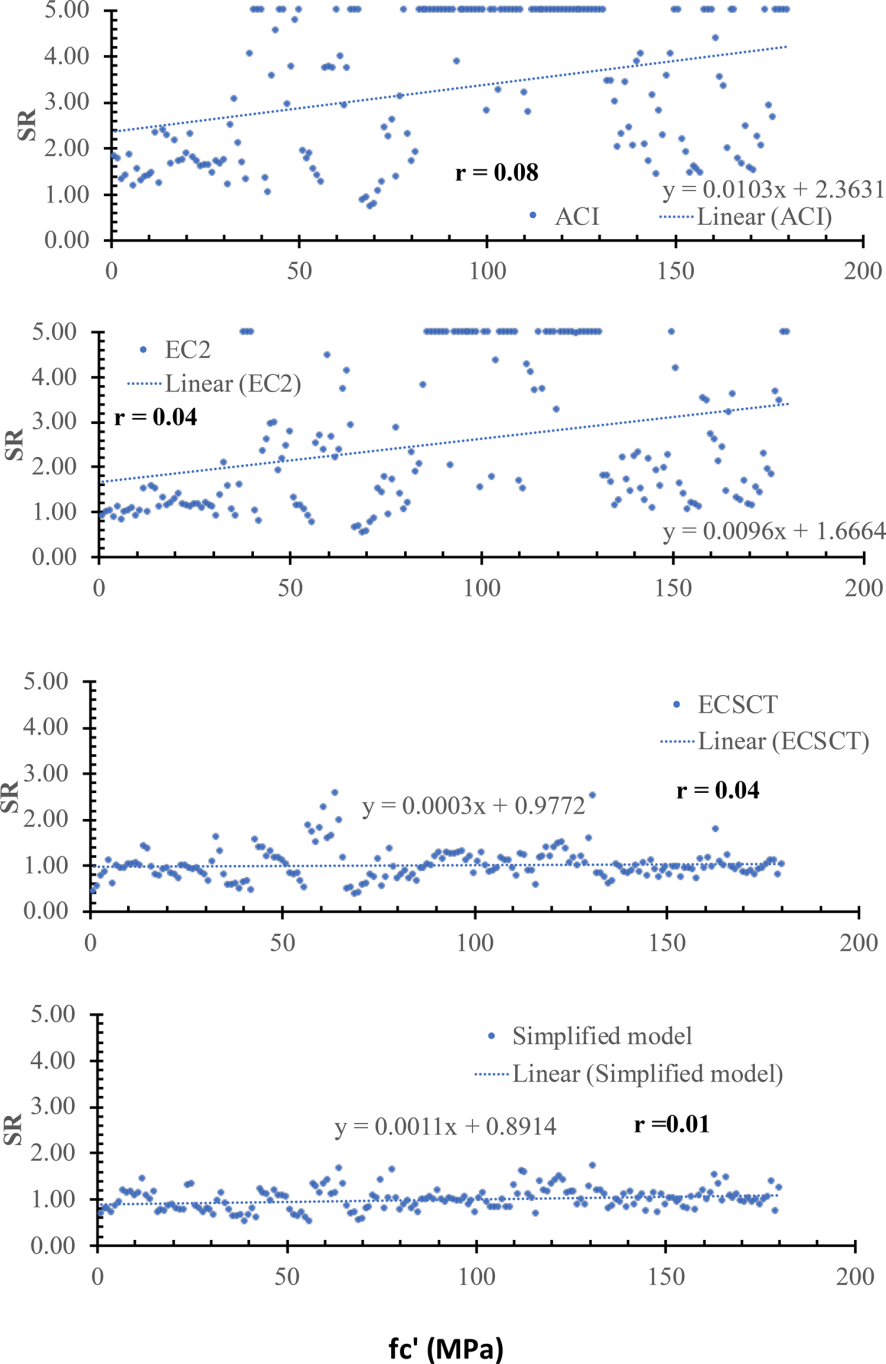
SR using various methods versus the fc′.

The SR is plotted versus the size in terms of the concrete compressive strength (*f*c′), as shown in Fig. 14. From Fig. 14, the safety of the strength calculated using the ECSCT and the simplified model is more consistent with the fc′ effect compared with the ACI and EC2. In addition, the correlation coefficient (r) was calculated as 0.08, 0.04, 0.04, and 0.01 for the ACI, the EC2, the ECSCT, and the simplified model, respectively. Thus, it is clear that SR calculated using the ACI, the EC2, the ECSCT, and the simplified model is weakly correlated to the *f*c′. Moreover, the slope of the best fit line for the SR calculated using the ACI, the EC2, the ECSCT, and the simplified model is 103E-4, 96E-4, 3E-4, and 11E-4, respectively. Thus, the slope for the SR calculated using the ECSCT and simplified model is significantly lower than that calculated using the ACI and the EC2. Therefore, it is clear that the ECSCT and the simplified model are more consistent and accurate with respect to the direct shear mechanism and the residual tensile stresses in terms of the concrete strength (*fc′*).

### Effect of width to depth ratio (${\varvec{b}}/{\varvec{d}}$)

**Figure 15:**
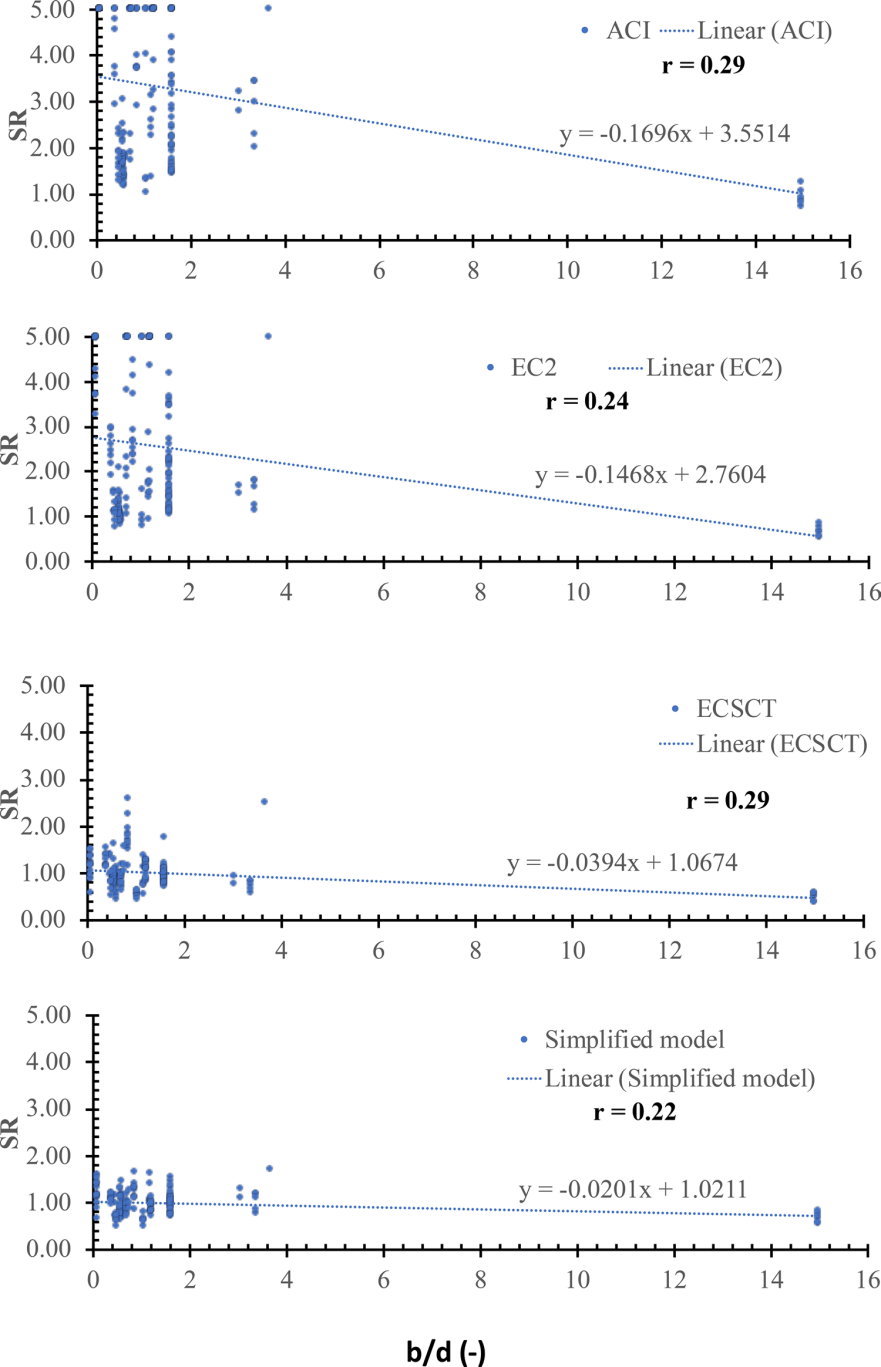
SR using various methods versus the b/d.

The SR is plotted versus the size in terms of the width to depth ratio ($\mathbf{b}/\mathbf{d}$), as shown in Fig. 15. From Fig. 15, the safety of the strength calculated using the ECSCT and the simplified model is more consistent with the effect of the ${\varvec{b}}/{\varvec{d}}$ compared with the ACI and EC2. In addition, the correlation coefficient (r) was calculated as 0.29, 0.24, 0.29, and 0.22 for the ACI, the EC2, the ECSCT, and the simplified model, respectively. Thus, it is clear that SR calculated using the ACI, the EC2, the ECSCT, and the simplified model is moderately correlated to the ${\varvec{b}}/{\varvec{d}}$. Moreover, the slope of the best fit line for the SR calculated using the ACI, the EC2, the ECSCT, and the simplified model is 17E-2, 15E-2, 4E-2, and 2E-2, respectively. The slope for the SR calculated using the ACI and the EC2 is significantly higher than that calculated using the ECSCT and simplified model. Therefore, it is clear that the ECSCT and the simplified model are more consistent with respect to the aspect ratio.

## Conclusions

A physically sound mechanical model capable of accurately reproducing the actual behavior of reinforced concrete members under combined shear and tension is proposed. In addition, the model is accurate and simple for design. The effect of axial tensile forces on the compression zone depth and longitudinal strain and, ultimately, on the shear strength is accounted for. The proposed model is based on the principles of mechanics and its applicability to reinforced concrete elements under shear combined with tension. In addition, a simplified model is proposed for the purpose of design. The two proposed models were found to be more accurate, consistent, and reasonably safe compared to selected design codes. Moreover, the effect of basic parameters on the safety of the proposed models and the selected design codes was assessed. For all basic variables, including (1) the axial tension; (2) the shear span to depth ratio; (3) the flexure reinforcement ratio; (4) the concrete compressive strength; and (5) the width to dept ratio, the following conclusions were reached.

- The correlation relation with the safety factor calculated using the American and European design code was very strong, while that for the proposed models was very weak. Thus, the proposed models captured the effect of all basic variables much better than the selected design models.
- The slope of the best fit line for the safety factor calculated using the proposed models is very small compared to that using the selected design codes. Thus, the proposed models are more consistent with the basic variables than existing design codes.

## Data Availability

All data generated or analysed during this study are included in this published article.
